# Online dashboards for SARS-CoV-2 wastewater data need standard best practices: An environmental health communication agenda

**DOI:** 10.2166/wh.2023.312

**Published:** 2023-05

**Authors:** Colleen C. Naughton, Rochelle H. Holm, Nancy J. Lin, Brooklyn P. James, Ted Smith

**Affiliations:** a Department of Civil and Environmental Engineering, University of California Merced, Merced, CA 95343, USA; b Christina Lee Brown Envirome Institute, School of Medicine, University of Louisville, Louisville, KY 40202, USA; c Material Measurement Laboratory, National Institute of Standards and Technology, Gaithersburg, MD 20899, USA

**Keywords:** COVID-19, health communication, sewage, standards, technology, wastewater-based epidemiology

## Abstract

The COVID-19 pandemic has highlighted the benefits of wastewater surveillance to supplement clinical data. Numerous online information dashboards have been rapidly, and typically independently, developed to communicate environmental surveillance data to public health officials and the public. In this study, we review dashboards presenting SARS-CoV-2 wastewater data and propose a path toward harmonization and improved risk communication. A list of 127 dashboards representing 27 countries was compiled. The variability was high and encompassed aspects including the graphics used for data presentation (e.g., line/bar graphs, maps, and tables), log versus linear scale, and 96 separate ways of labeling SARS-CoV-2 wastewater concentrations. Globally, dashboard presentations also differed by region. Approximately half of the dashboards presented clinical case data, and 25% presented variant monitoring. Only 30% of dashboards provided downloadable source data. While any single dashboard is likely useful in its own context and locality, the high variation across dashboards at best prevents optimal use of wastewater surveillance data on a broader geographical scale and at worst could lead to risk communication issues and the potential for public health miscommunication. There is a great opportunity to improve scientific communication through the adoption of uniform data presentation conventions, standards, and best practices in this field.

## Graphical Abstract

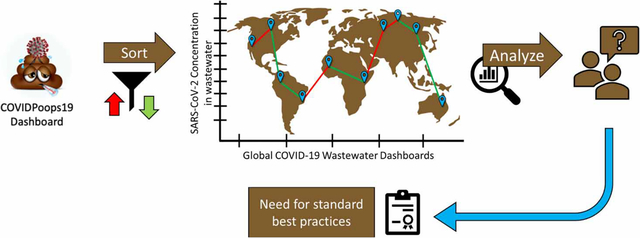

## BACKGROUND

Wastewater surveillance for severe acute respiratory syndrome coronavirus 2 (SARS-CoV-2) has been used since early in the coronavirus disease 2019 (COVID-19) pandemic to complement clinical testing (Medema et al. 2020; Wu et al. 2020; World Health Organization 2022a). A benefit of matched wastewater surveillance for a geographic area is that it is anonymous and captures both symptomatic and asymptomatic infections and removes some of the pandemic-related inequities in testing, positivity, and confirmed cases (Medema et al. 2020; Wu et al. 2020; Bilal et al. 2021). Surveys of public awareness and perceptions of wastewater surveillance have reported strong support for wastewater data (Holm et al. 2022a; LaJoie et al. 2022). To this end, online data dashboards can serve as a one-way message of wastewater results from researchers to both other experts and non-research audiences.

The current context for our research agenda is that risk communication problems may arise while establishing and recognizing credibility, making messages understandable, capturing and focusing attention, and obtaining data (National Research Council 1989). Stakeholders, including the World Health Organization (2022a, 2022b), have promoted online information dashboards for presenting SARS-CoV-2 wastewater viral concentrations. However, this task is not to be taken lightly, as the historical context of improper risk communication from environmental data from scientists has been evidenced. For example, failure to evaluate and communicate earthquake hazard data to the public resulted in fatalities and injuries, and scientists and a government official went on trial after the 2009 L’Aquila, Italy, earthquake (Hall 2011). The unclear scientific statements to the public during the active earthquake temporal window where alternative precautionary actions could have been taken are attributed as a key in the communication failure and resulted in devastating public consequences.

In the case of wastewater surveillance data during the COVID-19 pandemic, the rapid development of scientific communication approaches via online data dashboards has been conducted in the absence of a unified organizing body, despite efforts to convene the field to address this issue. For instance, the National Institute of Standards and Technology (NIST) and the Department of Homeland Security, Science and Technology Directorate (DHS S&T) co-hosted a workshop on standards for wastewater surveillance (SWWS) (Lin et al. 2022; Servetas et al. 2022). Their findings highlighted the need for standards to support the entire workflow of wastewater surveillance from sampling to the use of data, including communication. Among various standards, documentary standards are consensus-based documents that range from highly prescriptive performance requirements to general best practices. The SWWS workshop highlighted the lack of documentary standards to support the burgeoning field of wastewater surveillance. In the area of data reporting and analytics, workshop attendees indicated that the following documentary standards would have the greatest impact: standard template for reporting results, data standards, metadata requirements including quality assurance samples, and limits of detection (Lin et al. 2022). Guidance and best practices to support and facilitate consistency across online dashboards would improve the communication of wastewater surveillance results.

Herein, we reviewed 127 dashboards and evaluated their presentation formats for consistency of SARS-CoV-2 wastewater viral concentration across global online dashboards that seek to communicate public health risks. Additionally, we critically reflected on the standards and best practices needed to facilitate consistent online data communication.

## METHODS

The list of currently available public dashboards was compiled from COVIDPoops19 (COVIDPoops19 2022; Naughton et al. 2023) as of March 31, 2022. Google Translate was used for the non-English dashboards. Researchers visited each dashboard to tabulate data parameters. Due to the wide variation between dashboard presentation, relevant sub-links within the dashboards were checked for further information. This study was conducted before the United States Centers for Disease Control and Prevention (2022) National Wastewater Surveillance System public dashboard was established. To determine country-level income classifications, the World Bank (2022) gross national income per capita guidelines in 2020 were used.

### Ethics

The dashboards used in the analysis are public records available online, the links and spatial representation of which are provided in Supplementary Material, Files 1 and 2.

## RESULTS

Of the 127 dashboards identified, 125 were accessible at the time of this analysis (two websites that had been previously tracked had been moved or were offline). More than half (58%; 72/125) of the dashboards provided SARS-CoV-2 wastewater-paired COVID-19 clinical case data which allows a user to extract information on community risk from the dashboards for example on increasing/decreasing transmission levels or high-/medium-/low-risk levels. Only the presentation of wastewater data was evaluated within the scope of this study.

### Location of the wastewater surveillance programs with a dashboard

Twenty-seven countries had SARS-CoV-2 wastewater dashboards, 94% (118/125) of which were high-income countries. While some high-income countries had a single dashboard, others had many; the United States had 66 unique dashboards, Canada had 22, and Australia had five. The remaining dashboards (6%; 7/125) belonged to middle-income countries; of these, Brazil and South Africa had two unique dashboards, respectively. In terms of spatial representation, globally the dashboards included: East Asia and Pacific region at 5% (6/125); Europe and Central Asia region at 19% (24/125); Latin America and the Caribbean region at 2% (2/125); Middle East and North Africa region at 1% (1/125); North America region at 70% (88/125); South Asia region at 2% (2/125); and the sub-Saharan Africa region at 2% (2/125). Dashboards presented a range of territorial levels inclusive of city, state, and countrywide. Multiple dashboards within a country using different metrics suggest opportunities for standardization and best practices for risk communication.

### Units of measure

Data were presented in a range of formats, including line/bar graphs, maps, and tables with symbols. We identified 96 separate labeling descriptors for SARS-CoV-2 wastewater concentration, with many instances of synonymous terminology (Table 1). Of these, some form of quantitative (in contrast to presence/absence or trend observation) wastewater unit results were given in 85% (106/125) of dashboards. The most common was a derivative of gene copies/L or gene copies/mL (27%; 34/125). Differences in the units can be confusing. For example, the Erie County Pennsylvania (2023) dashboard conveyed a y-axis of ‘COVID-19 Amounts in Wastewater,’ which from a risk communication standpoint confuses the resulting disease with the indicator cause. This expression can come across as more reader-friendly but is not scientifically accurate.

For dashboards with units of measurement, normalization of the raw data for the presentation was also common (26%; 25/96) but with some regional characteristics noted. Population normalization was performed by dividing by the number of people served within the sampled sewer service area (for example, by 100,000 inhabitants) (16%; 15/96), less frequently by the fecal indicator pepper mild mottle virus (PMMoV) concentration (8%; 8/96), or by sewer system flow rate (2%; 2/96). Some dashboards, such as that by Healthy Davis Together (2023), had a non-numeric y-axis attributed to their normalization method. Two other dashboards displayed data in the form of exponentially weighted moving average (EWMA) per day, Bengaluru (Bangalore), India (Precision Health – Bangalore Response 2023) and Missouri, USA (The Sewershed Surveillance Project 2023), which again may be difficult for the public to interpret. There were 15 dashboards that presented some form of normalization by population size sampled. Out of these, nine were spatially represented in Europe and Central Asia, five were in North America, and one was in East Asia and the Pacific region. Of the eight dashboards that presented viral concentration normalized by PMMoV, six were in the North America region and two were in the Europe and Central Asia region. North America and Europe and Central Asia region dashboards were more likely to present normalization than other regions; North America dashboards tended to normalize wastewater concentration by PMMoV while Europe and Central Asia region dashboards tended to normalize by population size sampled.

### Scaling

**Figure 1 |:**
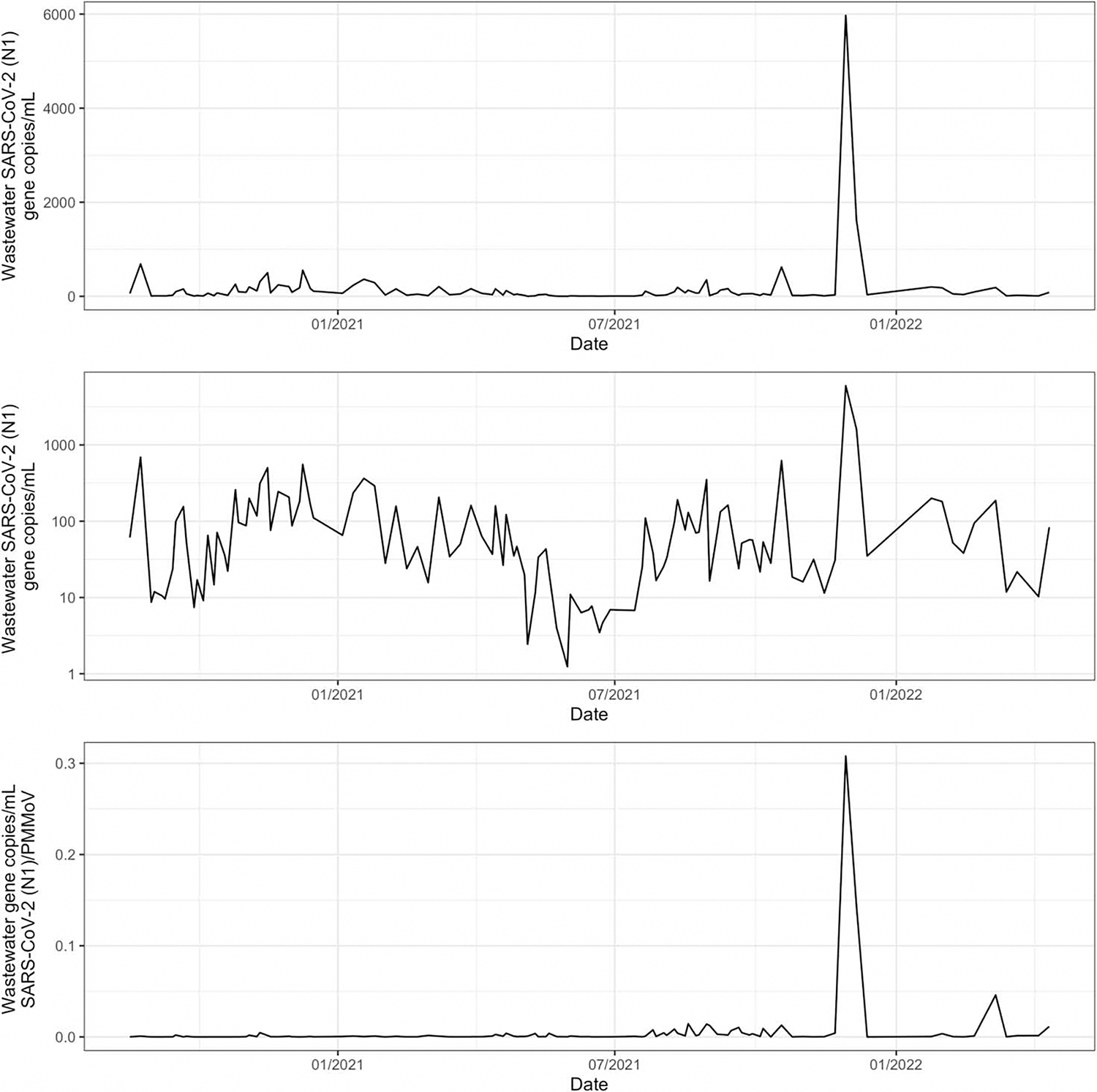
Using a single dataset, three simulated SARS-CoV-2 wastewater concentration time-series data presentations were prepared for August 2020–April 2022. Top panel displays wastewater concentration on a linear scale; middle panel displays wastewater concentration on a logarithmic scale; and bottom panel displays the same wastewater concentration divided by PMMoV concentration. The simulated SARS-CoV-2 wastewater concentration dataset is based on the earlier work by Holm et al. (2022b).

**Figure 2 |:**
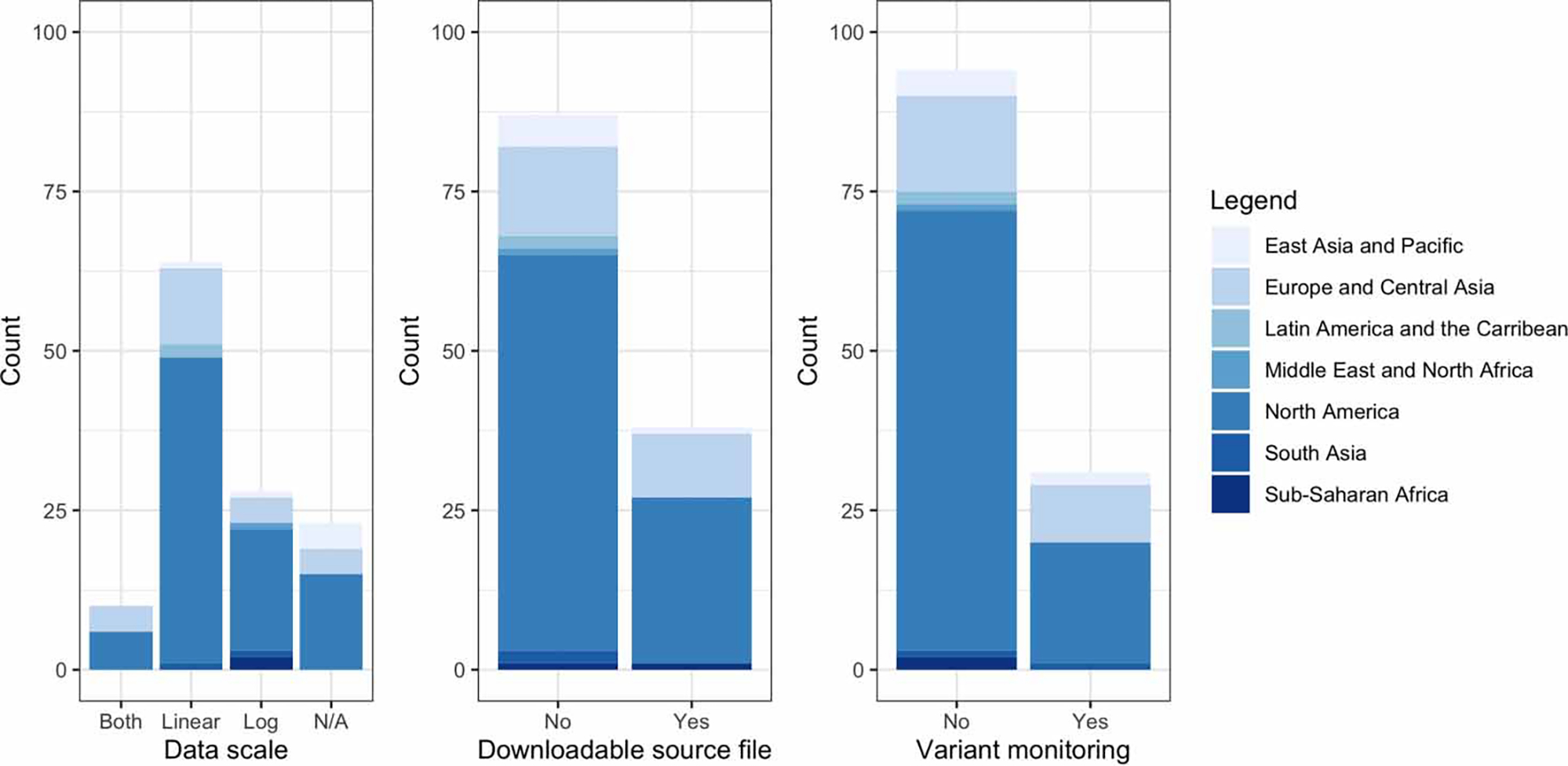
Variability across geographical regions in online dashboard presentation for SARS-CoV-2 wastewater at scaling including both log and linear scales, linear scale only, log scale only, or where scale was not applicable (left); whether the dashboard provided a downloadable source file for wastewater concentration data, or not (middle); and whether or not dashboards presented data on wastewater SARS-CoV-2 variant tracking (right). To determine geographical region classifications, the World Bank (2022) guidelines were used.

The scale of data is important for risk communication and dashboard user understanding of transmission levels. Where units were presented, a linear-only scale was observed more often (63%; 64/102) than a logarithmic scale (27%; 28/102). Ten dashboards (10%; 10/102) presented both logarithmic and linear scales (Figures 1 and 2). Some (34%; 35/102) dashboards with line/bar graphs had a double y-axis, commonly with COVID-19 clinical cases, hospitalizations, vaccine rates, or the number of deaths thoughtfully scaled with wastewater data. The wastewater concentration on the left y-axis and normalized wastewater data on the right y-axis were also presented in some dashboards.

The date was the most common scale of intersection, with 60 dashboards presenting data back to 2020, 36 back to 2021, and six starting in the first quarter of 2022. Not all dashboards used line or bar graphs. For example, two dashboards from Australia presented qualitative data in a table format for sample location by date, whereas the Canadian Water Network Wastewater Collection Maps uniquely presented a partnership dashboard on locations processing samples (public lab or university lab), although there was no wastewater data. One French dashboard stood out for straightforward 30-day trend map (rising, falling, or steady) observations represented through size and color scale disk variation (Sorbonne Université 2023).

### Variant monitoring

One quarter (25%; 31/125) of dashboards presented data on SARS-CoV-2 variant tracking, mostly (94%; 29/31) from high-income countries (Figure 2). Variant monitoring was typically displayed in a sub-link by comparing the percentage of each variant present. The variant tracking dashboard presentation ranged from using digital droplet polymerase chain reaction (ddPCR) assay results to present derived proportion estimates of variants (Switzerland) to presenting quantified results from genomic sequencing (Jefferson County, KY). In terms of spatial representation, variant monitoring globally on dashboards included: 19 in the North America region, nine in the Europe and Central Asia region, two in East Asia and the Pacific region, and one in the South Asia region.

### Data transparency

Although online dashboards can be viewed as a form of scientific communication, only 30% (38/125) provided a downloadable source file (Figure 2). These files ranged from quantifiable data as Microsoft Excel files in a variety of row and column formats to figures in a portable network graphic file format. In general, data transparency was low across the surveyed dashboards. In terms of spatial representation, downloadable source data available on dashboards included the following: 26 in the North America region, 10 in the Europe and Central Asia region, one in the East Asia and the Pacific region, and one in the sub-Saharan Africa region. While North America and Europe and Central Asia region dashboards were more likely to present downloadable source files, for each of these regions data transparency remained poor with less than half of dashboards providing this level of detail.

## DISCUSSION

The current landscape of data presentation for SARS-CoV-2 wastewater data through online dashboards demonstrates significant variation and presents an opportunity for improvement through documentary standards and best practices. Environmental data dashboards have been shown to be a positive method for engaging stakeholders and supporting water and electricity conservation behaviors, and a way to bridge academics and communities being surveyed (Petersen et al. 2017). For clinical COVID-19 samples, the real-time online dashboard of Johns Hopkins University (Baltimore, MD) is an example of individual diagnostic testing results aggregated from many global sources with a unified presentation (Dong Du & Gardner 2020). However, the principles for rapid and responsible data sharing in health outbreaks have been focused on clinical testing (Yozwiak et al. 2015) rather than environmental health analogs. Additionally, there are hundreds more sampling locations where wastewater data collection occurs without an online dashboard (Naughton et al. 2023), but standards should be applied globally. Variability in the way the data are normalized or presented across dashboards within a country could hamper the understanding of transmission dynamics within and across borders. For example, if members of the public traveling for work or vacation want to know about COVID-19 community spread patterns, they can easily look at clinical case data across geographic locations for comparable results, whereas looking across wastewater dashboards for another location is challenging and inconsistent.

We found few common dashboard themes, takeaways, or examples of how public health principles were woven. Even the different impressions of the scale should be emphasized (Figure 1; Romano et al. 2020; Sevi et al. 2020) where the doubling of SARS-CoV-2 wastewater values for trend analysis on a linear scale is muted on a log scale for larger values, though not for smaller values. This contrasts with other environmental surveillance data such as that of *Escherichia coli* in drinking water, whereby the World Health Organization (2022c) guideline is 0 colony-forming units (cfu)/100 mL, and the *y*-axis is most commonly in cfu/100 mL, but the *x*-axis could routinely be the date or sample location. In microbiology, log scales are the norm attributed to a dynamic range of multiplying organisms. Whereas *E. coli* drinking water data presentation scales focus on the attainment of the lower end of the WHO guideline of 0 cfu for the protection of human health, for SARS-CoV-2 the trends over time are more important to track spread patterns. Changing between a log and linear scale of a progressively dynamic range of COVID-19 metrics has been shown to alter the public’s perceptions and policy preferences (Romano et al. 2020), for the case of wastewater this similarly would likely also apply if visually comparing a different log/linear scale across dashboards even with the same units. For SARS-CoV-2 wastewater dashboards, having a log/linear-scale toggle is important.

We found that 85% of the studied dashboards provided a quantitative wastewater result, which contrasts with risk communication around polio detection in wastewater (World Health Organization 2003) where presence/absence is the level of detail required as a trigger of a possible outbreak. When SARS-CoV-2 quantitative data is presented in line/charts, we found the predominant *y*-axis was a derivative of gene copies/L or gene copies/mL against the *x*-axis of date; regardless, units of measure should be clearly stated. Normalized data may additionally be presented if metadata describing the dataset, such as the flow rate or a fecal indicator at the sample site, and calculations are also displayed.

Dashboards have already been meeting needs within communities, even as imperfect as they are; the great potential is limited due to the current high variation in data treatment. The extent of the data presentation problem may not be fully understood at present, as metrics beyond viral concentration are not widely available. The motivation for accurate hazard data communication becomes evident when reflecting on the poor hazard data communication from scientists and government officials before the 2009 earthquake in L’Aquila, Italy (Hall 2011). In the case of the COVID-19 pandemic, communication involves increasing/decreasing transmission levels or high-/medium-/low-risk levels. While SARS-CoV-2 viral concentration could be considered the basic level of data transparency, variant monitoring has evolved in pandemic data frameworks (Smith et al. 2022) as well as the importance of factoring in the acuity of the illness and impact on the healthcare delivery system. However, variant monitoring requires higher-level laboratory facilities that may not be available in all public health centers, and standardization may be more nuanced given variant monitoring is an evolving capability.

There are important limitations to this analysis. Some entities elect not to share data in a dashboard, because they do not have the resources to build one (hence they simply share numerical data in tables) or because they choose to limit the data access to public health professionals or decision-makers due to privacy and/or misinterpretation concerns. Disclosing the type of entities (public, private, academia) collecting the data should be made, and this was unclear in many instances of our review. As well, the majority of the dashboards reviewed in this article are based in the North America region. Further, our review focused on harmonization and improved risk communication, and we did not consider data presentation variability resulting from different laboratory methods, such as variability in primer and probes for quantitative polymerase chain reaction (qPCR) and ddPCR. Many dashboards did not describe their laboratory analysis methods.

The broader scientific community is better served to promote accuracy and reduce bias, even if the dashboard data are not comparable as displayed, if it is downloadable and allows users to be able to transform the data to make it more comparable. We recommend downloadable machine-readable data to support this mission. Provision of wastewater source data, aggregated and de-identified, should also adhere to FAIR (Findability, Accessibility, Interoperability, and Reusability) open data management standards to support machine-readable data, and many countries have signed open data agreements (Naughton et al. 2023). The standardization of units is one area where our results show wastewater surveillance data does not comply with FAIR. Dashboards require scientists to develop the data presentation, and the wealth of data available from wastewater surveillance will not be easily accessible via artificial intelligence without a common format for data presentation. These FAIR machine-readable metadata requirements for online data dashboards should include metadata addressing field and laboratory details such as the name of entity reporting the data, sample date, sample location, analysis date, analysis method, the limit of detection, what was analyzed, concentration, and units at a minimum (McClary-Gutierrez et al. 2021). Data presentation does not need to include only the presence and quantification of viral concentration; there is also value in indicating its absence (or a quantified result below detection). This area requires thoughtful global reporting standardization and consensus.

Key considerations for a new standard format for online dashboards presenting SARS-CoV-2 wastewater data include:

- Standards should apply globally.
- Units of measure should include the *y*-axis labeled as gene copies/L or gene copies/mL if liquid, and *x*-axis labeled as date, along with a toggle to switch between linear and log transformed data. If normalization is used, raw data should also be displayed.
- Metadata should be available covering both field and laboratory details, with disclosure of entity details.
- Source data should be machine-readable and downloadable.
- Dashboards should adhere to FAIR open data standards.
- It is never too late to start; researchers should continually be encouraged to add online dashboards.
- Consensus and knowledge sharing should be built among all stakeholders.

## CONCLUSION

We urge those at the forefront of the dashboards presenting SARS-CoV-2 wastewater concentrations to promote their standardization. Clear and consistent communication of wastewater results with non-research audiences and, specifically, the greater public, is urgently needed. We observed some strengths, such as presenting wastewater alongside clinical data for a geographical area, considering variants, and allowing log/linear-scale toggles. The clearest example from our review of the need for standardization in the current landscape is significant variation in units of measurement for SARS-CoV-2 concentration as well as different ways of labeling units with synonymous terminology and no clear precedent to follow; this is a significant issue and opportunity. For data transparency, not only is it not straightforward for non-science audiences to have such variation in units, but more importantly, it suggests that the underlying science still does not have a rationale for how viral concentration should be calculated and presented. Spatial characteristics were also observed; when viral concentration normalization was presented, the North America region dashboards tended to normalize by PMMoV whereas Europe and Central Asia region dashboards were more likely to normalize by population size sampled. Furthermore, potential work on consensus-building with all involved communities, utilities, and public health departments remains. Our environmental health agenda on data-driven standards and best practices can be useful for other wastewater-based epidemiological online dashboard applications as the field continues to expand beyond COVID-19. The ability to communicate SARS-CoV-2 wastewater concentrations effectively to non-scientific audiences, including journalists and policymakers, along with approaches to avoid miscommunication, is a critical gap. The capacity to incorporate wastewater surveillance data into public health actions is potentially hampered by inconsistencies in the way data are analyzed and presented, so a uniform standard is urgently needed.

## Supplementary Material

Supplementary Material

## Figures and Tables

**Table 1 |:** Select SARS-CoV-2 wastewater concentration unit groups as presented in online dashboards

Group	Example units^a,b^

Viral concentration	cp/mL COVID-19 amounts in wastewater genome copies/L genome copies/L of sewage genome copies/mL wastewater genome/L log10 gene copies/L log10 RNA Copies/L mean virus copies/L relative SARS-CoV-2 levels SARS-CoV-2 RNA copies/mL SARS-CoV-2 (weighted mean; gc/L) standardized concentration of SARS-CoV-2 Gene Copies RNA copies/mL virus copies/L wastewater viral load (Log10 Copies)
Viral concentration normalized by population size sampled	# virus particles/100,000 inhabitants copies SARS-CoV-2/100K gene copies/day/100,000 people log10 Copies/day/person M copies/person/day SARS-CoV-2 RNA copies/day/100,000 eq. inhabitants μg/L/100K inhabitants viral gene copies/person
Viral concentration normalized by time	average COVID million genes/day EWMA copies/day gc/day N1, N2, IP4 GC/day SARS-CoV-2 RNA flux (copies/day)
Viral concentration normalized by a fecal indicator	concentration of SARS-CoV-2/PMMoV copies/mL normalized to PMMoV copies/mL ×1,000 copies/PMMoV N1 gene copy number to PMMoV (×10^4^) normalized viral copies (to PMMoV) normalized viral copies (*μ*) (by PMMoV) PMMoV normalized N1 (×1,000) ratio of gene N1 and PMMoV

a The example units listed here are taken directly from the dashboard websites. There is substantial variation in the units and abbreviations used, and many of these do not comply with SI unit rules.

b cp, copies; gc and GC, genome copies; M, million; EWMA, exponentially weighted moving average; N1, N2, and IP4 refer to target genes in the SARS-CoV-2 genome; PMMoV, pepper mild mottle virus.

## Data Availability

All relevant data are included in the paper or its Supplementary Information.

## HIGHLIGHTS

- Rapid development of presenting SARS-CoV-2 wastewater data via online dashboards.
- Reviewed 127 global SARS-CoV-2 wastewater dashboards and evaluated presentation formats.
- Dashboard variability was high across data presentation, line/bar graph scales, downloadable source files, units of measure, and variant monitoring.
- Opportunity to adopt uniform data presentation conventions, standards, and best practices in this field.
