# Supplementary material for: Online dashboards for SARS-CoV-2 wastewater data need standard best practices: An environmental health communication agenda
Source: J Water Health. Author manuscript; Available in PMC 2024 Feb 6. (PMC10846915; doi:10.2166/wh.2023.312)
Supplement: Supplementary Material [file NIHMS1960367-supplement-Supplementary_Material.docx]

**SUPPLEMENTARY FILE 1**

List of online dashboards for SARS-COV-2 wastewater from COVIDPoops19 website as of March 31, 2022. To determine country level income classifications, the World Bank (2022) gross national income per capita guidelines in 2020 were used: low-income economies (<$1,085), lower middle-income economies ($1,086 to $4,255), upper middle-income economies ($4,256 to $13,205), and high-income economies ($13,205+). Spatial representation was also by World Bank (2022) regions.

**East Asia and Pacific**

| **Web link** | **Country** | **World Bank income category** |
| --- | --- | --- |
| <https://www.dhhs.vic.gov.au/wastewater-monitoring-covid-19> | Australia | High income |
| <https://www.qld.gov.au/health/conditions/health-alerts/coronavirus-covid-19/current-status/wastewater> | Australia | High income |
| <https://www.health.nsw.gov.au/Infectious/covid-19/Pages/sewage-surveillance.aspx> | Australia | High income |
| <https://www.coronavirus.tas.gov.au/facts/covid-19-wastewater-testing/covid-19-wastewater-testing-results> | Australia | High income |
| <https://ww2.health.wa.gov.au/Articles/A_E/Coronavirus/COVID19-wastewater-testing> | Australia | High income |
| <https://www.esr.cri.nz/our-expertise/covid-19-response/wastewater-testing-results/> | New Zealand | High income |

**Europe and Central Asia**

| **Web link** | **Country** | **World Bank income category** |
| --- | --- | --- |
| <https://www.coron-a.at/dashboard/> | Austria | High income |
| <https://corona.hydro-it.com/> | Austria | High income |
| <https://datastudio.google.com/embed/u/0/reporting/c14a5cfc-cab7-4812-848c-0369173148ab/page/p_ggbfgsqtmc> | Belgium | High income |
| <https://covid-pulse.cy/> | Cyprus | High income |
| <https://covid19.ssi.dk/overvagningsdata/overvaagning-af-sarscov2-i-spildevand> | Denmark | High income |
| <https://www.thl.fi/episeuranta/jatevesi/jatevesiseuranta_viikkoraportti.html> | Finland | High income |
| <https://www.reseau-obepine.fr/carte-des-tendances/> | France | High income |
| <https://covidtracker.fr/covidexplorer/> | France | High income |
| <https://bimsbstatic.mdc-berlin.de/akalin/AAkalin_pathogenomics/sarscov2_ww_reports/210528_wastewaterall_pigxsarscov2ww_default/index.html> | Germany | High income |
| <http://trams.chem.uoa.gr/covid-19/> | Greece | High income |
| <https://www.nnk.gov.hu/index.php/koronavirus/szennyvizvizsgalatok> | Hungary | High income |
| <https://bior.lv/lv/par-mums/jaunumi/notekudenu-monitorings-covid-19-izplatibas-noteiksanai> | Latvia | High income |
| <https://www.list.lu/en/covid-19/coronastep/> | Luxembourg | High income |
| <https://coronadashboard.rijksoverheid.nl/landelijk/rioolwater> | Netherlands | High income |
| <https://covid-19.sledilnik.org/en/stats> | Slovenia | High income |
| [https://miteco.maps.arcgis.com/apps/opsdashboard/index.html#/a8c1f281dfc445169a78178b70774a62](https://miteco.maps.arcgis.com/apps/opsdashboard/index.html#/a8c1f281dfc445169a78178b70774a62 ) | Spain | High income |
| <https://edarbens.es/covid19/> | Spain | High income |
| <https://sarsaigua.icra.cat/> | Spain | High income |
| <https://www.canaldeisabelsegunda.es/mapa-vigia> | Spain | High income |
| <https://crush-covid.shinyapps.io/crush_covid/> | Sweden | High income |
| <https://covid19dataportal.se/data_types/environment/wastewater/> | Sweden | High income |
| <https://www.eawag.ch/en/department/sww/projects/sars-cov2-in-wastewater/> | Switzerland | High income |
| <https://covid19.tarimorman.gov.tr/> | Turkey | Upper-middle |
| <https://informatics.sepa.org.uk/RNAmonitoring/> | United Kingdom | High income |

**Latin America and the Caribbean**

| **Web link** | **Country** | **World Bank income category** |
| --- | --- | --- |
| <https://t.co/RY4D877qeW?amp=1> | Brazil | Upper-middle |
| <https://app.powerbi.com/view?r=eyJrIjoiNzMxYjdiZGYtZDVjNy00NTMwLWIwZmItYmQwOWJhNzk3YmU1IiwidCI6Ijc1NmU3MTc4LTA1ZmYtNGVmYy05OTY2LTU2ODFlNjE2MjA3MCJ9&pageName=ReportSectiond497bb36400a320db4c7> | Brazil | Upper-middle |

**Middle East and North Africa**

| **Web link** | **Country** | **World Bank income category** |
| --- | --- | --- |
| <https://app.powerbi.com/view?r=eyJrIjoiNjY5ZjM2ZTctMGM5Mi00NTZmLTk0NjMtZWQ2OThkYWZhMTA1IiwidCI6ImIzYzdlZDM0LWQxZjAtNDg5Zi05YzllLWE0YmNlYTk0YmJlNCIsImMiOjl9> | Israel | High income |

**North America**

| **Web link** | **Country** | **World Bank income category** |
| --- | --- | --- |
| [https://613covid.ca/wastewater/#](https://613covid.ca/wastewater/) | Canada | High income |
| <https://wechu.org/cv/weekly-epidemiological-summary> | Canada | High income |
| <https://covid-tracker.chi-csm.ca/> | Canada | High income |
| <https://nwt-covid.shinyapps.io/Testing-and-Cases/?lang=1> | Canada | High income |
| <http://www.metrovancouver.org/services/liquid-waste/environmental-management/covid-19-wastewater/Pages/default.aspx> | Canada | High income |
| <https://www.kflaph.ca/en/healthy-living/covid-19-in-city-of-kingston-wastewater.aspx> | Canada | High income |
| <http://www.519covid.ca/> | Canada | High income |
| [https://www.peelregion.ca/health-professionals/covid-19/#symptoms](https://www.peelregion.ca/health-professionals/covid-19/#symptoms ) | Canada | High income |
| [https://water.usask.ca/covid-19/#ResearchersandFunding](https://water.usask.ca/covid-19/#ResearchersandFunding ) | Canada | High income |
| <https://www.centreau.ulaval.ca/donnees-du-projet-centreau-covid/> | Canada | High income |
| [https://www.regionofwaterloo.ca/en/health-and-wellness/covid-19-wastewater-surveillance.aspx#](https://www.regionofwaterloo.ca/en/health-and-wellness/covid-19-wastewater-surveillance.aspx# ) | Canada | High income |
| <https://cwn-rce.ca/covid-19-wastewater-coalition/covid-19-wastewater-coalition-maps/> | Canada | High income |
| <https://app.powerbi.com/view?r=eyJrIjoiMjU2MmEzM2QtNDliNS00ZmIxLWI5MzYtOTU0NTI1YmU5MjQ2IiwidCI6IjUyZDdjOWMyLWQ1NDktNDFiNi05YjFmLTlkYTE5OGRjM2YxNiJ9> | Canada | High income |
| <https://www.simcoemuskokahealthstats.org/topics/infectious-diseases/a-h/covid-19/covid-19-wastewater-surveillance> | Canada | High income |
| <https://news.uoguelph.ca/2019-novel-coronavirus-information/u-of-g-covid-19-wastewater-report/> | Canada | High income |
| <https://www.york.ca/wps/portal/yorkhome/health/yr/covid-19/covid19inyorkregion/01covid19inyorkregion/!ut/p/z1/tZJLT-MwFIV_C4suI187SW0vTeg0CTQtjz7iTZVJ09RMk5SMKTC_fhxUJISgMGLshV-6Plfn80ESLZCss70qM62aOtuacyr7y0gMozA8h3jssQAEjEVMKIMBx2j-XAAfDAFIfuX9kQJ5XH6GJJK7XK1QStyCspwzh4KfO16W9x2Of67MxNc-5-uMd3JI5rXe6Q1Kn9pl3tS6qHUPnpr2lzn81krfP19smqowc5Ft9aYHebNXKwfzww5zVXcv2qI0mHoA-J1rFH_m3cAl7SgYlcZBpjeOqtcNWrz0OuzeiC7e72Wk1O3dnRTGXufpUaOFbX_zDuZrh8Nr5kE0i6mY4TF4kXsoIMTrhziAGMIxg-gHnfhnLMRwTg4FR_43NfmgH0K8Imi-V8UDmtZNW5m8Xv9jHMKXDpQFIhRDmMDNlMLlgHqsfzGaXFzhb3b4xIBledeqPAW78sSu_P-BE0cQYNHF3x24IEgUsFM3Zklil31il31il31iN_ez78LZVdNpxVx_WzLNo1u_rJZnp4mTxvs_R5eRODn5Cx0G6fA!/dz/d5/L2dBISEvZ0FBIS9nQSEh/#.Yd3nnYjMK3A?utm_source=newmarkettoday.ca&utm_campaign=newmarkettoday.ca%3A%20outbound&utm_medium=referral> | Canada | High income |
| [https://covid19-sciencetable.ca/ontario-dashboard/#wastewatersignal](https://covid19-sciencetable.ca/ontario-dashboard/#wastewatersignal ) | Canada | High income |
| <https://healthunit.org/health-information/covid-19/local-cases-and-statistics/dashboard/> | Canada | High income |
| <https://www.toronto.ca/home/covid-19/covid-19-pandemic-data/covid-19-wastewater-surveillance/> | Canada | High income |
| <https://www.tbdhu.com/coviddata> | Canada | High income |
| <https://www.gov.nl.ca/ecc/waterres/wastewater-surveillance-for-covid-19-virus/> | Canada | High income |
| <https://www.wesparkhealth.com/covid-screening-platform#Dashboard> | Canada | High income |
| <https://data.covid-web.org/> | United States | High income |
| <https://lipplab-uga.github.io/covid_wastewater_lipplab_athens/> | United States | High income |
| <https://yalecovidwastewater.com/> | United States | High income |
| <https://coronavirus.ohio.gov/wps/portal/gov/covid-19/dashboards/wastewater> | United States | High income |
| <https://techimpact.shinyapps.io/ncco_wastewater/> | United States | High income |
| <https://www.cityofboise.org/departments/mayor/coronavirus-covid-19-information/covid-19-data/wastewater-testing/> | United States | High income |
| <https://www.healthygallatin.org/coronavirus-covid-19/wastewater-data/> | United States | High income |
| <https://experience.arcgis.com/experience/19a447509ff44fcf922ad0892b7b2cce/page/page_21/> | United States | High income |
| <https://www.indianaboro.com/news/categories/wastewater-surveillance> | United States | High income |
| <https://nantucket-ma.gov/1864/Surfside-Wastewater-Treatment-Facility-C> | United States | High income |
| <https://www.keene.edu/featured/fall2020/covid-19-dashboard/> | United States | High income |
| <https://www.burlingtonvt.gov/covid-19/wastewater> | United States | High income |
| <https://public.tableau.com/profile/oregon.health.authority.covid.19#!/vizhome/OregonsSARS-CoV-2WastewaterMonitoring/WastewaterDashboard> | United States | High income |
| <https://loxahatcheeriver.org/wastewater-surveillance/> | United States | High income |
| <https://www.dhs.wisconsin.gov/covid-19/wastewater.htm> | United States | High income |
| <https://eriecountypa.gov/tag/biobot/> | United States | High income |
| <https://egle.maps.arcgis.com/apps/webappviewer/index.html?id=8e3cb66aca204876a1db0e1b663af805> | United States | High income |
| <https://connect.chattanooga.gov/covid-biobot-analysis-reports/> | United States | High income |
| <https://www.cor.pa.gov/Pages/COVID-19.aspx> | United States | High income |
| <https://www.hrsd.com/HRSD-COVID-19-Surveillance> | United States | High income |
| [https://cdphe.maps.arcgis.com/apps/opsdashboard/index.html#/d79cf93c3938470ca4bcc4823328946b](https://cdphe.maps.arcgis.com/apps/opsdashboard/index.html#/d79cf93c3938470ca4bcc4823328946b ) | United States | High income |
| <https://storymaps.arcgis.com/stories/f7f5492486114da6b5d6fdc07f81aacf> | United States | High income |
| <https://cityofcambridge.shinyapps.io/COVID19/#shiny-tab-wastewater> | United States | High income |
| <https://www.env.nm.gov/wastewater-surveillance-system-data-dashboard/> | United States | High income |
| <https://returntolearn.ucsd.edu/dashboard/index.html> | United States | High income |
| <https://www.lccountymt.gov/health/covid-19/local-covid-19-decision-making-dashboard.html> | United States | High income |
| <https://covid-testing.uconn.edu/dashboard/> | United States | High income |
| [https://sanmiguelco.maps.arcgis.com/apps/opsdashboard/index.html#/56e682135d1d4128bee1a0426aed1d10](https://sanmiguelco.maps.arcgis.com/apps/opsdashboard/index.html#/56e682135d1d4128bee1a0426aed1d10 ) | United States | High income |
| <http://smchd.org/covid-19-wastewater> | United States | High income |
| <https://soe-wbe-pilot.wl.r.appspot.com/charts#page=overview> | United States | High income |
| <https://healthydavistogether.org/wastewater-testing/> | United States | High income |
| <https://southhadley.org/DocumentCenter/Index/604> | United States | High income |
| <https://coronavirus.berkeley.edu/dashboard/wastewater/> | United States | High income |
| <https://www.sccgov.org/sites/covid19/Pages/dashboard-wastewater.aspx> | United States | High income |
| <https://trace.oregonstate.edu/testing-results> | United States | High income |
| <https://arcg.is/1KySSv> | United States | High income |
| <https://www.palmspringsca.gov/government/departments/community-economic-development-department/wastewater-treatment-plant-covid-19-test-reports> | United States | High income |
| <https://wastewater.covid19.mathematica.org/> | United States | High income |
| <https://louisville.edu/envirome/thecoimmunityproject/dashboard> | United States | High income |
| <https://covid19.ncdhhs.gov/dashboard/wastewater-monitoring> | United States | High income |
| <http://erie.gov/covid19-wastewater/> | United States | High income |
| <https://covidwastewatermonitor.wyo.gov/> | United States | High income |
| <https://experience.arcgis.com/experience/4456bae8a38f4b6180e008477382fff9/page/page_0/> | United States | High income |
| <https://covidwwtp.spatialstudieslab.org/> | United States | High income |
| <https://datastudio.google.com/u/0/reporting/430e67c8-acaf-4574-a2d4-48d0b665ab05/page/jMhOC> | United States | High income |
| <https://www.michigan.gov/coronavirus/0,9753,7-406-98163_98173-573480--,00.html> | United States | High income |
| <https://umich-wbe.shinyapps.io/sars-cov-2_dash/> | United States | High income |
| <https://mdewin64.mde.state.md.us/WSA/SSI/index.html> | United States | High income |
| <https://metrotransitmn.shinyapps.io/metc-wastewater-covid-monitor/> | United States | High income |
| [https://mbcolli.shinyapps.io/SARS2EWSP/#](https://mbcolli.shinyapps.io/SARS2EWSP/) | United States | High income |
| <https://www.district2framingham.com/2022/01/20/framingham-covid-19-update/> | United States | High income |
| <https://engineering.utsa.edu/vkapoor/research/wastewater-covid/> | United States | High income |
| <https://www.lsu.edu/roadmap/covid-dashboard/index.php> | United States | High income |
| <https://www.ubcleanwater.org/pretreatment/pages/covid-19-wastewater-monitoring> | United States | High income |
| [https://covid.cdc.gov/covid-data-tracker/#wastewater-surveillance](https://covid.cdc.gov/covid-data-tracker/#wastewater-surveillance ) | United States | High income |
| <https://www.cdph.ca.gov/Programs/CID/DCDC/Pages/COVID-19/CalSuWers-Dashboard.aspx> | United States | High income |
| <https://www.maine.gov/dhhs/mecdc/infectious-disease/epi/airborne/coronavirus/data.shtml> | United States | High income |
| <https://experience.arcgis.com/experience/a8d269bd670a421e9fd45f967f23f13c> | United States | High income |
| <https://www.tulsa-health.org/coronavirus-disease-2019-covid-19/tulsa-county-covid-19-data> | United States | High income |
| <https://thenevadaindependent.com/coronavirus-data-nevada> | United States | High income |
| [https://publichealth.verily.com/#Sunnyvale,%20TX](https://publichealth.verily.com/#Sunnyvale,%20TX ) | United States | High income |
| <https://www.clemson.edu/covid-19/testing/wastewater-dashboard.html> | United States | High income |
| <https://deq.utah.gov/water-quality/sars-cov-2-sewage-monitoring> | United States | High income |
| <https://covid19.tempe.gov/> | United States | High income |
| <http://www.mwra.com/biobot/biobotdata.htm> | United States | High income |
| [https://portal.rjngroup.com/arcgisportal/apps/opsdashboard/index.html#/594d4b1b2dd840958cedb50b1381982b](https://portal.rjngroup.com/arcgisportal/apps/opsdashboard/index.html#/594d4b1b2dd840958cedb50b1381982b ) | United States | High income |

**South Asia**

| **Web link** | **Country** | **World Bank income category** |
| --- | --- | --- |
| <https://erin-wettstone.shinyapps.io/Dashboard_V6/> | Bangladesh | Lower-Middle |
| <https://storymaps.arcgis.com/stories/c42be68c85634d19a5d92873a10bda66> | India | Lower-Middle |

**Sub-Saharan Africa**

| **Web link** | **Country** | **World Bank income category** |
| --- | --- | --- |
| <https://www.samrc.ac.za/wbe/> | South Africa | Upper-middle |
| <https://www.nicd.ac.za/diseases-a-z-index/disease-index-covid-19/surveillance-reports/weekly-reports/wastewater-based-epidemiology-for-sars-cov-2-in-south-africa/> | South Africa | Upper-middle |

Reference

World Bank 2022 *World Bank country and lending groups*. https://datahelpdesk.worldbank.org/knowledgebase/articles/906519-world-bank-country-and-lending-groups (accessed 20 April 2022).

**SUPPLEMENTARY FILE 2**

List of online dashboards for SARS-CoV-2 wastewater data covering scaling, variant monitoring, and data transparency by World Bank (2022) regions. To determine country level income classifications, the World Bank (2022) gross national income per capita guidelines in 2020 were used: low-income economies (<$1,085), lower middle-income economies ($1,086 to $4,255), upper middle-income economies ($4,256 to $13,205), and high-income economies ($13,205+). Spatial representation was also by World Bank (2022) regions.

**Scaling**

| **World Bank region** | **Observed scale** | | | | |
| --- | --- | --- | --- | --- | --- |
|  | **Both Linear and Log** | **Linear** | **Log** | **N/A** | **Total** |
| East Asia and Pacific |  | 1 | 1 | 4 | 6 |
| Europe and Central Asia | 4 | 12 | 4 | 4 | 24 |
| Latin America and the Caribbean |  | 2 |  |  | 2 |
| Middle East and North Africa |  |  | 1 |  | 1 |
| North America | 6 | 48 | 19 | 15 | 88 |
| South Asia |  | 1 | 1 |  | 2 |
| Sub-Saharan Africa |  |  | 2 |  | 2 |
| **Total** | **10** | **64** | **28** | **23** | **125** |

**Variant monitoring**

|  | **Was data presented on SARS-CoV-2 variant tracking?** | | |
| --- | --- | --- | --- |
| **World Bank region** | **No** | **Yes** | **Total** |
| East Asia and Pacific | 4 | 2 | 6 |
| Europe and Central Asia | 15 | 9 | 24 |
| Latin America and the Caribbean | 2 |  | 2 |
| Middle East and North Africa | 1 |  | 1 |
| North America | 69 | 19 | 88 |
| South Asia | 1 | 1 | 2 |
| Sub-Saharan Africa | 2 |  | 2 |
| **Total** | **94** | **31** | **125** |

**Data transparency**

| **Count of downloadable data** | **Was downloadable source file available?** | | |
| --- | --- | --- | --- |
| **Row labels** | **No** | **Yes** | **Total** |
| East Asia and Pacific | 5 | 1 | 6 |
| Europe and Central Asia | 14 | 10 | 24 |
| Latin America and the Caribbean | 2 |  | 2 |
| Middle East and North Africa | 1 |  | 1 |
| North America | 62 | 26 | 88 |
| South Asia | 2 |  | 2 |
| Sub-Saharan Africa | 1 | 1 | 2 |
| **Total** | **87** | **38** | **125** |

**REFERENCE**

World Bank 2022 *World Bank country and lending groups*. https://datahelpdesk.worldbank.org/knowledgebase/articles/906519-world-bank-country-and-lending-groups (accessed 20 April 2022).
